# Effects of 3D Contemplative Landscape Videos on Brain Activity in a Passive Exposure EEG Experiment

**DOI:** 10.3389/fpsyt.2018.00317

**Published:** 2018-07-30

**Authors:** Agnieszka A. Olszewska-Guizzo, Tiago O. Paiva, Fernando Barbosa

**Affiliations:** ^1^School of Design and Environment, National University of Singapore, Singapore, Singapore; ^2^Faculty of Psychology and Education Sciences, University of Porto, Porto, Portugal

**Keywords:** contemplative, landscape, ART, design, well-being, mental health, EEG

## Abstract

**Background:** Studies on Contemplative Landscapes (CL) show that not only do they have high ecological and visual values and are preferred by a majority of people, but they also can be beneficial to our mental health and well-being. Physical attributes of CL have been studied and operationalized, which has led to the development of the psychometric measurement tool called the Contemplative Landscape Questionnaire (CLQ) (1).

**Objectives:** In the present study, we applied an experimental approach to the analysis of CL. We hypothesized that, when compared to Non-Contemplative Landscapes (NCL), they would induce higher frontal alpha power asymmetry, higher temporal beta power asymmetry and lower bilateral frontal beta power.

**Methods:** Thirty-two healthy individuals (12 female) took part in the study. During the experimental protocol, participants were asked to passively view 12 landscapes, six CL and six NCL, while continuous EEG was recorded in a within-subjects design.

**Results:** We found significantly increased power in the beta frequency band of the right temporal brain regions in the viewings of CL compared to NCL

**Conclusions:** The findings suggest that Contemplative Landscapes capture more visual, stimuli-driven attention from the viewers and can be linked with switching attention systems (described in Attention Restoration Theory), which is compatible with a stress reduction mechanism.

## Introduction

Current trends in landscape architecture and urban planning show the increasing demand for space design solutions that will achieve both environmental and social benefits. Therefore, the concept of Evidence-Based Design (EBD) has emerged, attracting increasingly more attention of researchers and designers, as well as decision makers (2–5). The research attempts, within the EBD, to emphasize credible evidence to inform design of green open spaces, especially in the urban context, to improve people's health and well-being. This emerging field includes, among other areas of expertise, the methods of psychology and neuroscience.

Accordingly, our experiment aims at examining the potential differences in brain activity patterns between the processing of landscapes identified as Contemplative (CL) and Non-Contemplative Landscapes (NCL). CL are green outdoor settings, which, according to the CLQ, are characterized by a combination of multiple features (long vistas, lush seemingly-wild vegetation, the presence of symbolic elements, and smooth landforms, among others) and scored above 4.5 points in a 1-6 point scale, which means the explicit accumulation of the key features (1). NCL, on the other hand, are green outdoor settings that scored lower than 3.0 points on the same scale. The construct of CL may be linked with the Attention Restoration Theory (ART) with a so-called restorative effect of exposure to natural environments, which can reduce stress and mental fatigue, improve positive emotions and promote a sensation of well-being (6–8). These effects may be indirectly linked with the therapeutical benefits of natural environments in the treatment of psychiatric issues such as bipolar disorder and ADHD, among others; however, there is no enough evidence on that yet (9). Moreover, ART did not attempt to specify and systematize physical attributes of restorative environments, while the CL approach focuses on specific features of the green outdoor landscapes that may trigger these effects.

Environmental psychologists set the concept of attention restoration against the concepts of stress and mental fatigue (7, 8). Accordingly, research shows that the relaxing benefits of natural landscapes can stimulate the patterns of brain activity associated with positive emotional states (10, 11). One of such states is characterized by lower alpha power on the left frontal lobe in comparison to the right. This pattern would be followed with lower bilateral frontal beta power [e.g., (12, 13)]. Another possible state is greater beta activity in the right temporal regions as an index of visual attention (14, 15) and a bottom-up, stimulus-driven attention (16).

In the context of the present experiment, we hypothesized that CL would induce higher frontal alpha power asymmetry, higher temporal beta power asymmetry and lower bilateral frontal beta power, when compared to NCL. This could suggest that being exposed to CL may induce the pattern associated with positive emotional states and involuntary, effortless attention, as predicted by ART.

## Methods

### Participants

Thirty-two participants (12 female) were recruited among students and researchers of the University of Porto and took part in the study. Twenty-six were Portuguese, four Polish, one Russian, one American, and one Greek. The mean age of the participants was 27 y.o. (*SD* = 6.5 y.o.). All had completed at least 12 years of formal education. Four participants were left-handed and their recordings were included as this would not significantly change the results of the experiment.

All participants had normal, or corrected to normal, vision. None reported psychiatric, neurological or cognitive diseases. Also, none of the participants reported use of medication that could alter the functioning of the Central Nervous System at the time of the experiment. The existence of a pacemaker, intracranial electrodes, implanted defibrillator or plates, otologic surgery in the last 12 months, or any dentures, were additional exclusion criteria. None of the recruited participants was excluded. All participants signed the informed consent form and filled in the socio-demographic questionnaire.

### Materials

#### Stimuli

Twelve 3D fixed-angle videos representing landscape images that were previously rated by experts (landscape architecture and urban ecology academics), based on the CLQ (1) including six CL and six NCL videos. Each of the videos was 20 s long.

The recorded video format was TS(AVC)(“^*^.m2ts”) with a frame size of 1920 × 1080 pixels, 50i frame speed and 16:9 aspect ratio, recorded with a Sony™ HDR-TD30 camcorder. The videos were converted, using the Magix™ Movie Edit Pro 2015 software, to the mp4 file format with side-by-side composition of images, preserving the initial image resolution and aspect ratio. The individual landscape videos were mounted into two single video presentations of 2 min each, in order to obtain a block design protocol. The videos were displayed using the free software Bino 3-D video player, in the Left/Right and Open GL mode. This allowed the display of the entire set of videos for each condition by the projector (DepthQ HDs3D-1) on the screen (2,440 × 1,800 mm). To see the depth of the image, each subject wore Nvidia 3D shutter glasses, which were connected to the PC and received the infrared signal by the IR 3D emitter.

#### EEG apparatus

The EEG data were collected using an eight-channel electroencephalographic amplifier, Enobio model with dry electrodes, placed on a neoprene headcap. The device is wireless (operating with Bluetooth) with a Li-Ion battery, and connected to a laptop through the NIC 1.2 software (17).

### Procedure

#### Data collection

Data acquisition took place in a quiet room on the university premises. All electronic devices in the room were switched off, except the stimulation and EEG recording systems, to reduce all possible external electromagnetic artifacts. The experiment was prepared in a block design, where subjects were instructed to passively observe the videos of the selected landscapes. The videos were organized in two blocks—CL and NCL—counterbalanced across participants to control for order effects and displayed while their EEG activity was being recorded. Baseline recordings (BL) of 3 min were performed for each subject in resting state (looking at the empty screen with eyes open) before displaying the blocks of stimuli. Event markers at the beginning of the BL and at the onset of each new landscape were manually inserted in the EEG recordings.

The scalp of the participant was cleaned with a cotton-pad wetted with ethylic alcohol, with special attention to the areas where the electrodes were placed. Eight active dry electrodes were placed on the Enobio cap according to the International Position System 10–20 at the AF7, AF8, Fz, T7, Cz, T8, P3, and P4 referenced to an electrode placed at the right mastoid (RM). The electrode locations were selected according to the areas of interest that were identified in the literature (12–14). Electrode impedances were checked and kept below 20 kΩ at all sites, which is considered an acceptable value for dry electrodes (18, 19). The EEG signal was acquired in the 0–250 Hz band at a 500 Hz sampling rate. Line noise interference was reduced using a notch filter of 50 Hz.

Subjects were seated on a fixed chair, placed 2 m in front of the screen. The 3D shutter glasses were put on the participants' eyes without interfering with the cap, cables, or electrodes. In the case of subjects wearing correction glasses, they were kept under the 3D shutter glasses.

**Figure 1:**
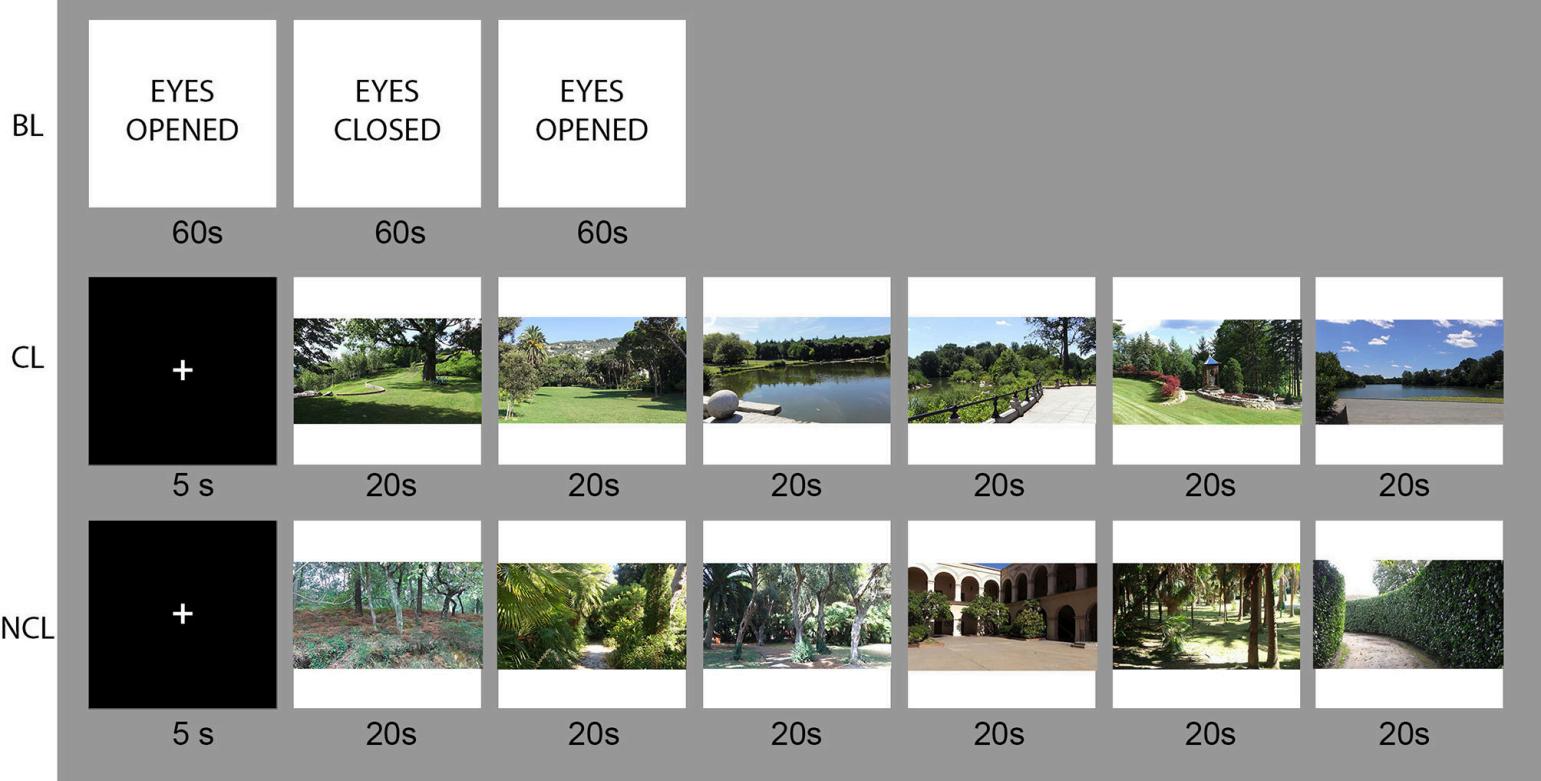
Task design and stimulus presentation for the resting state (BL), contemplative condition (CL), and non-contemplative condition (NCL); photo credits A. Olszewska-Guizzo.

Subjects were instructed to passively watch the videos of each landscape. Each subject was also informed about the three parts of the experiment and their duration: baseline recording at the beginning and CL/NCL landscapes presented in a counterbalanced order, with a fixation cross of 5 s before the beginning of each block. A schematic of the data recording procedure is displayed in Figure 1.

Subjects were instructed to sit still on the chair, avoid head, and body movements, and to focus the eye gaze on the center of the screen to prevent eye movement artifacts. They were also instructed to slightly open the mouth and reduce blinking as much as possible to diminish muscle artifacts.

#### Data processing and analysis

Data analysis was performed using MATLAB 5.3 with EEGLAB toolbox (20). The recorded EEG data was downsampled to 250 Hz and re-referenced to the average of all electrodes.

Data was then segmented into epochs of 20 s corresponding to each landscape for CL and NCL conditions. Regarding BL, a segment of 20 s was extracted between 120 and 140 s after the beginning of the recording to ensure the same duration of the segments.

After visual inspection, none of the segments was excluded due to defective signal quality.

Band-pass filters were then applied to all segments, targeting the frequencies of interest: alpha (7.5–14 Hz) and beta (14–30 Hz). As a result, we obtained two files per subject and per condition, each containing one of the frequencies described above. The mean absolute power was computed for each electrode as the average of the square voltage amplitude at each time point (μV^2^). Power values were log transformed in order to normalize their distributions, and asymmetries between brain hemispheres per EEG band were computed by applying the formula log(R)-log(L), where R is the power of a particular EEG band on the right hemisphere and L is the power on the left hemisphere. This procedure has been widely used in studies on asymmetries [e.g., (12, 21)]. The score is zero (no asymmetry) when left and right power is equal, whereas increased right over left activity results in higher scores (22). In sum, and in order to address our hypotheses, we obtained measures for: asymmetry between right and left hemisphere for alpha and beta frequency bands at the frontal and temporal electrode locations; and also, mean absolute power for beta frequency band at the frontal electrode locations (AF7 and AF8).

#### Statistical analysis

Two separated Repeated Measures ANOVA were conducted with *Condition* as within-subjects factor (BL, CL, NCL) and alpha frontal (AF7, AF8) asymmetry and beta temporal (T7, T8) asymmetry as dependent variables. A two-way Repeated Measures ANOVA with *Condition* and *Electrode Site* (AF7, AF8) as within-subjects factors and mean frontal beta power as dependent variable. Post-hoc multiple comparison tests were performed using the Holm-Sidak method (23) when the effect of condition was statistically significant (α = 0.05). Mauchly's tests indicated no sphericity violations, as their results were not significant (all *p* > 0.05).

## Results

### Frontal alpha asymmetry

Repeated-measures ANOVA revealed that the experimental *Condition* had no significant effect on frontal alpha asymmetry, *F*_(2, 62)_ = 1.51, *p* = 0.229, η^2^ = 0.81. The result remains insignificant after rejecting left-handed: *F*_(2, 54)_ = 2.51, *p* = 0.090, η^2^ = 0.80.

### Temporal beta asymmetry

Repeated-measures ANOVA revealed that the experimental *Condition* had a significant effect on temporal beta asymmetry, *F*_(2, 62)_ = 24.2, *p* < 0.001, η^2^ = 0.72. The differences in the mean values of temporal beta asymmetry between the resting state (*M*_BL_ = −0.016, *SD* = 0.208), when the subjects were viewing the Contemplative Landscapes (*M*_CL_ = 0.156, *SD* = 0.199) and when they were viewing the Non-Contemplative Landscapes (*M*_NCL_ = 0.093, *SD* = 0.167) were greater than what would be expected by chance and *post-hoc* analyses indicated significant differences between all pairs: NCL and BL (*p* < 0.001), CL and NCL (*p* = 0.015) and between CL and BL (*p* < 0.001).

**Figure 2:**
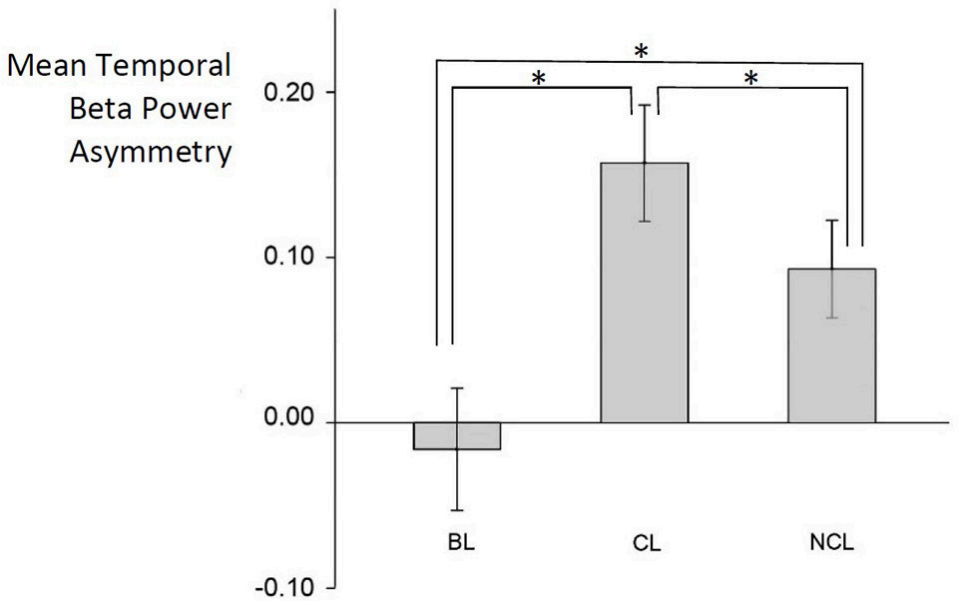
Differences in temporal beta asymmetry between resting state (BL), contemplative (CL), and non- contemplative (NCL) conditions. *symbol signifies *p* < 0.001.

As mean beta power asymmetry > 0, participants showed higher beta power on the right temporal side of the brain than the left temporal side (Figure 2).

These results remained similarly significant after rejection of left-handed participants: *F*_(2, 54)_ = 20.4, *p* < 0.001, η^2^ = 0.71. Also, post-hoc analyses revealed significant differences between all pairs of conditions: NCL and BL (*p* < 0.001), CL and NCL (*p* = 0.024) and between CL and BL (*p* < 0.001).

### Mean frontal beta power

The Two–Way repeated measures ANOVA revealed a main effect of *Electrode Site, F*_(1, 31)_ = 249.7, *p* < 0.001, η^2^*electrode* = 0.59, with lower frontal beta power on the AF7 (*M* = −0.035, *SD* = 0.271) than on the AF8 electrode (*M* = 0.627, *SD* = 0.283). A main effect was also found of *Condition, F*_(2, 62)_ = 205.3, *p* < 0.001, η^2^*Condition* = 0.16, with higher frontal beta power for BL (*M*_BL_ = 0.543, *SD* = 0.368), whereas subjects showed similar values for CL and NCL (*M*_CL_ = 0.165, *SD* = 0.425; *M*_NCL_ = 0.181, *SD* = 0.397). The interaction between *Electrode Site* and *Condition* revealed no effect, *F*_(2, 62)_ = 2.59, *p* = 0.082.

The *post-hoc* analyses using the Holm-Sidak method revealed that there was no significant difference between the CL and NCL condition, on both AF7 (*p* = 0.183) and AF8 electrodes (*p* = 0.806). On the contrary, the test revealed that the frontal beta power was significantly different on both AF7 and AF8 electrodes when comparing BL with CL and NCL conditions (both *p* < 0.001).

Rejecting left-handed participants did not change these results significantly*: Electrode Site F*_(1, 27)_ = 226.6, *p* < 0.001, η^2^*electrode* = 0.57; *Condition, F*_(2, 54)_ = 159.7, *p* < 0.001, η^2^*condition* = 0.17.

## Discussion

This study builds on the previously operationalized Contemplative Landscape Model (1). We intended to examine the effects of CL and NCL on brain activation patterns using experimental neurophysiology. We hypothesized that CL would induce lower alpha power in the left hemisphere, higher beta power on the right temporal regions and lower bilateral frontal beta would induce higher frontal alpha power asymmetry, higher temporal beta power asymmetry and lower bilateral frontal beta power, when compared to the NCL.

Our results revealed two main findings that support one of the hypotheses: we found greater beta power on the right temporal lobe, as expected, but we found no effects of condition on alpha power asymmetry, contrary to our prediction. Regarding frontal beta power, we found higher values of beta power on the baseline and no differences between CL and NCL landscapes, as we would expect.

Considering the temporal beta power asymmetry, our finding suggests a general activation of the right temporal areas of the brain while watching both CL and NCL scenes when compared to the baseline. This pattern was significantly stronger in the case of CL when compared to NCL, which may suggest that participants were more intensely perceiving the holistic aspect of the displayed landscapes, recognizing, and processing the general spatial relationships between its elements, e.g., perceiving a forest rather than a single tree. This brain activity pattern could also be associated with bottom-up, stimuli driven attention directed at the salient stimuli, which can be linked to ‘fascination’ (a key-component of restorative environment) (6, 16).

The right temporal areas of the brain are, among other functions, responsible for global visual attention (14, 15), visual information interpretation and memory of pictures, visual scenes, and familiar faces (24). The difference between CL and NCL suggests that contemplative landscapes capture more of this attention pattern from the viewers.

According to the Attention Restoration Theory (ART), attention consists of two components: “involuntary attention, where attention is captured by inherently intriguing or important stimuli, and voluntary or directed attention, where attention is directed by cognitive-control processes” (6). Directed attention includes all the tasks requiring mental effort, and after some time it induces mental fatigue in people. Attention may be “restored” by switching to involuntary attention, which is associated with exposure to natural environments. A similar model of attention mechanisms was presented by Corbetta and colleagues as goal-directed rather than stimulus driven attention. The latter is a bottom-up system oriented at perception of behaviourally relevant stimuli, which works as a “circuit breaker” for the top-down attention system (16). It seems that the pattern of brain activity induced by both CL and NCL in this experiment (the greater right temporal beta activity) can be associated with the involuntary attention mechanism, but in the case of CL scenes, this effect was more intense.

Regarding frontal alpha power asymmetry and frontal bilateral beta power, although our hypotheses were not confirmed, further research should address these issues, and thus extend the amount of research-based evidence on the effects of the visual quality of landscapes on brain activity. The presented study represents the first attempt to examine how the CL affect neural activation patterns. As an exploratory study, it has some limitations that future studies should consider, i.e., the sample size, although optimal for most typical EEG studies, can be considered relatively small given the low signal-to-noise ratio present in the recordings. Also, further studies could be conducted in natural environments despite the challenges posed by the level of background noise present in such environments.

Findings such as the ones in this study, linking neuroscience to landscape architecture, may shed light on the influences of landscape design on brain and mental states, with potential benefits for mental health and well-being. As an interface research field, studies crossing neuroscience and architecture will face several methodological challenges, but experiments like this one are important to pave the way.

## Ethics statement

The presented work is part of the PhD research conducted in accordance with the Guide for Research Ethics Committee Members available at https://sigarra.up.pt/up. The research project has been approved by the Scientific Council of the Faculty of Science at University of Porto.

All participants signed an informed consent form and detailed participant information sheets that described the aims, methods, and implications of the research, the nature of the participation and any benefits, risks, or discomfort that might ensue; explicitly stated that participation is voluntary and that anyone has the right to refuse to participate and to withdraw their participation or data at any time—without any consequences; stated how data will be collected and protected during the project and either destroyed or reused subsequently; stated what procedures will be implemented in the event of unexpected or incidental findings (in particular, whether the participants have the right to know, or not to know, about any such findings).

The personal data collected were coded, only researchers in charge of the study were able to identify the participant by the name. All personal data were destroyed after 28 days from the end of the data analysis.

## Author contributions

All three authors contributed substantially to the above work, with specific tasks. The main author (AO-G): conceptualization, methodology, investigation, validation, writing, original draft. Second author (TP): software; formal analysis, data curation, writing-review, and editing. Third author (FB): validation, supervision, writing-review, and editing.

### Conflict of interest statement

The authors declare that the research was conducted in the absence of any commercial or financial relationships that could be construed as a potential conflict of interest.
